# Outdoor Air Pollution and Depression in Canada: A Population-Based Cross-Sectional Study from 2011 to 2016

**DOI:** 10.3390/ijerph18052450

**Published:** 2021-03-02

**Authors:** Ashley K. Dores, Gordon H. Fick, Frank P. MacMaster, Jeanne V. A. Williams, Andrew G. M. Bulloch, Scott B. Patten

**Affiliations:** 1 Department of Community Health Sciences, Cumming School of Medicine, University of Calgary, 3280 Hospital Drive NW, Calgary, AB T2N 4Z6, Canada; akdores@ucalgary.ca (A.K.D.); ghfick@ucalgary.ca (G.H.F.); jvawilli@ucalgary.ca (J.V.A.W.); bulloch@ucalgary.ca (A.G.M.B.); 2 Department of Psychiatry and Pediatrics, Cumming School of Medicine, University of Calgary, 3280 Hospital Drive NW, Calgary, AB T2N 4Z6, Canada; fmacmast@ucalgary.ca; 3 Mathison Centre for Mental Health Research & Education and the Hotchkiss Brain Institute Cumming School of Medicine, University of Calgary, 3280 Hospital Drive NW, Calgary, AB T2N 4Z6, Canada; 4 Cuthbertson & Fischer Chair in Pediatric Mental Health, Alberta Children’s Hospital Research Institute, University of Calgary, 3280 Hospital Drive NW, Calgary, AB T2N 4Z6, Canada

**Keywords:** outdoor air pollution, major depressive episode, depressive symptoms, mental health, environmental health

## Abstract

To assess whether exposure to increased levels of outdoor air pollution is associated with psychological depression, six annual iterations of the Canadian Community Health Survey (*n* ≈ 127,050) were used to estimate the prevalence of a major depressive episode (2011–2014) or severity of depressive symptoms (2015–2016). Survey data were linked with outdoor air pollution data obtained from the Canadian Urban Environmental Health Research Consortium, with outdoor air pollution represented by fine particulate matter ≤2.5 micrometers (μm) in diameter (PM_2.5_), ozone (O_3_), sulfur dioxide (SO_2_), and nitrogen dioxide (NO_2_). Log-binomial models were used to estimate the association between outdoor air pollution and depression, and included adjustment for age, sex, marital status, income, education, employment status, urban versus rural households, cigarette smoking, and chronic illness. No evidence of associations for either depression outcomes were found. Given the generally low levels of outdoor air pollution in Canada, these findings should be generalized with caution. It is possible that a meaningful association with major depression may be observed in regions of the world where the levels of outdoor air pollution are greater, or during high pollution events over brief time intervals. Future research is needed to replicate these findings and to further investigate these associations in other regions and populations.

## 1. Introduction

Outdoor air quality is a leading environmental determinant of the global burden of disease [1,2]. It has been established that outdoor air pollution is associated with respiratory conditions (e.g., asthma [3], and chronic obstructive pulmonary disease (COPD) [4]), cardiovascular disease [5], lung cancer [6], and may increase susceptibility to lower respiratory infections [7]. Some emerging research suggests that outdoor air pollution may adversely impact mental health, particularly depression [8,9,10].

Outdoor air pollution is mainly a mixture of particulate matter (e.g., fine particulate matter ≤2.5 micrometers (µm) in diameter, PM_2.5_), and gaseous molecules (e.g., ozone, O_3_, sulfur dioxide, SO_2_, and nitrogen dioxide, NO_2_) [11]. Prior evidence indicates that air pollution acts as an environmental source of oxidative stress resulting in inflammation involving the central nervous system (CNS) [12,13]. These studies observed that exposure to increased levels of outdoor air pollution was associated with increased production of pro-inflammatory cytokines [14], neuroinflammation (e.g., glial cell activation [15]), and reduced dopamine production [16], both in humans and in animal models [17,18]. In particular, depression has increasingly been recognized as being influenced by inflammation, with functional imaging showing glial cell activation during depression [19,20,21,22]. The inhalation of increased levels of air pollution may lead to a reduction in the production of dopamine [15], a possible neuropathological mechanism linking outdoor air pollution to clinically relevant depression [23,24].

However, a complete understanding of the pathophysiology of major depression is still yet to be fully elucidated. More than 300 million people worldwide are affected by depression [25], and approximately 1 in 5 individuals will experience a major depressive episode (MDE) at some point during their life [26]. Considering depression is multiply determined, identification of all potential determinants is important for treatment and prevention [27,28,29]. Therefore, with the existence of plausible biological mechanisms, outdoor air pollution may be a modifiable environmental determinant of major depression [30,31]. Although interest regarding this association has been increasing, epidemiological evidence on the major depression-outdoor air pollution association is limited and inconsistent.

Some studies have previously reported a positive association between exposure to increased levels of PM_2.5_, O_3_, SO_2_, and NO_2_ with various indicators of depression [8,9,32,33,34], and depressive symptoms [30,31,35,36]. Other research has reported no evidence of an association [37,38,39]. A majority of the studies in the peer-reviewed literature have relied on indirect measures of major depressive disorder (i.e., emergency department visits [9,10,32,33,34,40], and self-reported clinician diagnosis of major depression [8,36,37]), and the symptoms of depression (i.e., self-reported depressive symptoms [8], and self-reported use of antidepressants [37]). Fewer studies have included measures of both major depression and depressive symptoms [8,36]. Despite the different range of outdoor air pollution levels in these studies, there remains no known threshold level of effect of air pollution on mental health. Consequently, there remains uncertainty about whether poor outdoor air quality is associated with depression.

To address these gaps in knowledge, the objective of this study was to assess whether exposure to increased levels of PM_2.5_, O_3_, SO_2_, and NO_2_ were associated with a greater prevalence of MDE or elevated severity of depressive symptoms.

## 2. Materials and Methods

### 2.1. Canadian Community Health Survey (Study Population)

This study used data from the annual Canadian Community Health Survey (CCHS) conducted between 2011 to 2016 [41]. The CCHS is a cross-sectional survey that collects information on overall health and its determinants from the Canadian household population aged 12 years old and older. It includes both core content and content that can be chosen by provinces. The target population consisted of household residents, which excluded full time members of the Canadian Armed Forces, individuals living on reserves or other aboriginal settlements, children living in foster care, the institutionalized, and individuals living in certain remote regions of Quebec. Collectively, the exclusions represent less than 3% of the Canadian household population each year [41].

A stratified multi-stage sampling procedure was used to select respondents to participate in each survey year of the CCHS. Households were selected from health regions, and then individuals were randomly selected based on the number of eligible respondents living in the residence when sampling occurred. Total response rates (product of household and respondent rates) ranged from 69.8% in 2011 to 61.3% in 2016. Statistics Canada provided sampling weights and bootstrap replicate weights to account for the unequal selection probabilities and to adjust for non-response [41]. Respondents were eligible for inclusion in the current analysis if they had completed the depression module (*n* ≈ 127,050). Since not all provinces and territories opted to retain the depression module in each year of the CCHS, the sample size of this module varied by survey year.

### 2.2. Measures of Major Depression

Major depression was assessed by the annual CCHS in two ways. First, the past 12-month prevalence of MDE from 2011 to 2014 was measured using the Short Form Composite International Diagnostic Interview (CIDI-SF) for major depression. This is a fully structured diagnostic interview that produces a predictive probability of MDE. A 90% or greater predicted probability was considered indicative of past-year MDE. Interpreted in this way, the CIDI-SF has 89.6% sensitivity, and 93.9% specificity compared to the Composite International Diagnostic Interview [42].

Second, the past 2-week prevalence of elevated depressive symptoms (2015–2016) was measured using the 9-item Patient Health Questionnaire (PHQ-9). This 9-item scale is a screening tool that assesses the severity of the symptoms of major depression, aligning with the syndromic definition of MDE in the DSM-5 (88% sensitivity, and 88% specificity) [43]. Responses include “not at all”, “several days”, “more than half the days”, and “nearly every day”. When used in screening depressive symptoms, scores of 10+ (moderate/severe) out of 27 indicate a need for further assessment [43].

### 2.3. Measures of Outdoor Air Pollution (Exposures)

Outdoor air pollution data were obtained from the Canadian Urban Environmental Health Research Consortium (CANUE), a research group providing a data platform for environmental exposures measured at the postal code-level in Canada (for information on CANUE outdoor air pollutant data see website (accessed 1 March 2021): https://www.canuedata.ca/metadata.php). All outdoor air pollution estimates were linked to 6-digit residential postal-codes using DMTI Spatial Inc. [44]. In Canada, one side of a city block or large apartment building represented a given postal code.

Four outdoor air pollutants (PM_2.5_, O_3_, SO_2_, and NO_2_) were available from CANUE and represented outdoor air pollution in this study. We first used annual average concentrations of PM_2.5_ in micrograms per cubic meter (µg/m^3^) based on tri-annual running averages (current year plus previous 2 years) [45]. These estimates were derived from combining ground-based monitoring estimates (obtained from the National Air Pollution Surveillance program) with satellite-derived PM_2.5_ (retrieved from NASA remote sensing satellites: MODIS, MISR, and SeaWIFS), and simulated near-surface estimates of PM_2.5_ (generated from the GEOS-Chem chemical transport model) [44,45,46,47]. Geographically weighted regression was used to predict the bias between the satellite-derived and simulated estimates of PM_2.5_ utilizing a 1 km spatial resolution grid pattern and validated using 10-fold validation without replacement [48]. Despite the limited number of ground-based monitoring stations, this multifaceted approach is believed to accurately provide a continuous geospatial distribution of PM_2.5_ [47].

Second, to assess exposure to O_3_, we used annual average concentrations of near-surface O_3_ in parts per billion (ppb) estimated by Environment and Climate Change Canada (ECCC) [44,49]. Hourly ground-based measurements were used in the Global Environmental Multi-scale Modelling Air Quality and Chemistry (GEM-MACH) model with a spatial resolution of 10 km to produce a continuous geospatial distribution of O_3_ [50,51].

Third, CANUE reports annual average concentrations of SO_2_ in ppb based on tri-annual running averages (current year and previous 2 years) estimated by ECCC from satellite remote sensing data retrieved from the Ozone Monitoring Instrument, located on NASA’s AURA satellite [52,53]. The satellite-derived estimates were used in the GEM-MACH model with a 20 km spatial resolution grid pattern to produce continuous geospatial distribution of SO_2_ [44,52].

Fourth, annual average concentrations of NO_2_ in ppb were estimated by CANUE using land use regression (LUR) models from 2006 National Air Pollution Surveillance monitoring data [54,55,56]. The LUR models included satellite-derived estimates of the regional and background components of NO_2_ levels in Canada and deterministic gradients modeled local variation. The final LUR model included: road length within 10 km, 2005–2011 satellite-derived estimates of NO_2_, area of industrial land use within 2 km, and summer rainfall.

### 2.4. Neighborhood-Level Socioeconomic Status

Environmental measures of neighborhood-level deprivation were expected to demonstrate a spatial pattern and were, therefore, included to provide context to the main analysis. We used both of the social deprivation (SD) and material deprivation (MD) sections of the 2016 Material and Social Deprivation Index (MSDI) data, also obtained from CANUE [44,57]. The MSDI data were taken from the Pampalon Index that uses 6 indicators of neighborhood-level socioeconomic status from the 2016 Canadian Census on the (1) proportion of divorced, widowed, or single adults; (2) proportion of single-parent families; (3) proportion of individuals living alone; (4) average income; (5) proportion of adults without a high school diploma or equivalent; and (6) the ratio of employment to population, age, and sex standardized to represent the Canadian population aged 15 years old and older [58]. Marital status, and the indicators of family structure defined SD, and income, education, and employment status indicators defined MD. The social and material deprivation scores were also indexed to 6-digit postal codes via DMTI Spatial Inc. and deprivation estimates were divided into quintiles from least deprived (quintile 1) to most deprived (quintile 5).

### 2.5. Measures of Covariates

Age, sex, marital status, total household income, education, employment status, urban versus rural households, cigarette smoking, and chronic illness were assessed with standard Statistics Canada field-tested interview questions. Age of respondent was further classified into youth (12–24 years old) and adults (25+ years old), and then age as a continuous variable centered at 15 years old was used in the adjusted models (minimum age of respondents in adjusted models). Total household income was classified into four quartiles (quartile 1 as lowest). Chronic illness was a composite variable identifying respondents reporting a diagnosis by a health professional of at least 1 of 10 possible chronic illnesses (arthritis, asthma, back problems, cardiovascular disease, chronic bronchitis, COPD, diabetes, high blood pressure, migraine, and stroke). The nine potential covariates were selected as they are all considered individual-level determinants of major depression [26,59].

### 2.6. Statistical Analyses

**Figure A1:**
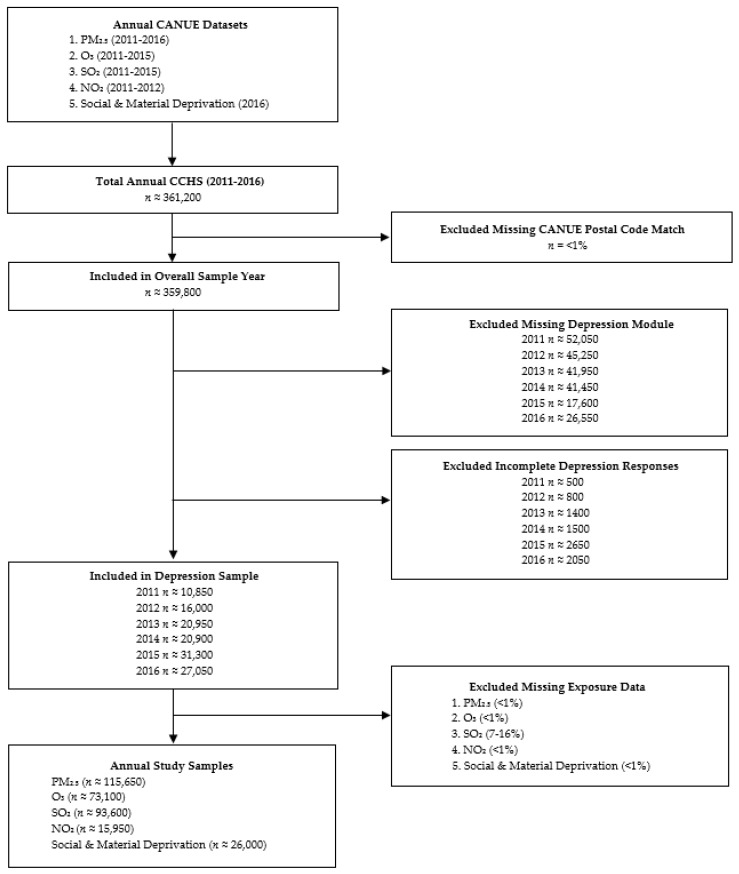
Flow chart of the annual sample sizes. Sample sizes are rounded to base 50 as per Statistics Canada guidelines and may not add up.

Figure A1 in Appendix A is a flow chart describing the method used to generate the annual study samples. Continuous outdoor air pollution levels were classified into quartiles, similarly to the definition reported in Shin et al. 2018 [8]. In the absence of an empirical cut-point, the upper concentration quartile of each outdoor air pollutant defined exposure to increased levels of outdoor air pollution in Canada each year. Annual sample years were population weighted and representative of the Canadian household population. Respondents were excluded from this study if they had incomplete responses on the depression module, were proxy interviews (no attempt was made to assess depression by proxy), or they were missing CANUE data. Most of the missing CCHS data from non-response was due to proxy interviews, with <1% of respondents having 1 or more missing answers to the depression questions from the CIDI-SF (2011–2014), or the PHQ-9 (2015–2016) questionnaires. Respondents excluded for missing outdoor air pollution data was primarily in the SO_2_ analysis (where CANUE did not have estimates for 7–16% of postal codes), with the rest of the missing outdoor air pollution data representing <1% of the annual sample populations.

Statistical significance was determined at α = 0.05 for all analyses. We used log-binomial regression models using the prevalence ratio (PR) as the measure of association between exposure to increased levels of outdoor air pollution and depression [60]. Potential modification (by examination of interaction terms), and then potential confounding were considered through forward selection. In the absence of modification, potential confounding was concluded to be present based on a judgement of meaningful change, guided by the convention of using a 10% change in the estimates of the crude PR and the PR with adjustment (aPR). In the absence of observed modification or confounding, variables were retained in the model to allow reporting of the adjusted estimates. Stata version 16.0 [61] was used to carry out all data analyses.

## 3. Results

A description of the study samples and estimates of the annual average concentrations of PM_2.5_, O_3_, SO_2_, and NO_2_ from 2011 to 2016 are presented in Supplementary Table S1, and Figure S1, respectively. Estimates of the demographic characteristics in 2016 are reported as percentages with corresponding 95% confidence intervals (95% CIs) in Table 1 (2011–2015 data were not shown as they were similar). As expected, a greater proportion of respondents with elevated severity of depressive symptoms in 2016 were younger (12–24 years old), female, and within the lowest total household income quartile (Table 1). This pattern was also observed for both depression measures and in all sample years. The other demographic characteristics, such as mean age, sex, marital status, education, and employment status remained consistent between exposure groups for all four outdoor air pollutants across their respective sample years (2016 PM_2.5_ data shown in Table 1).

**Figure 1:**
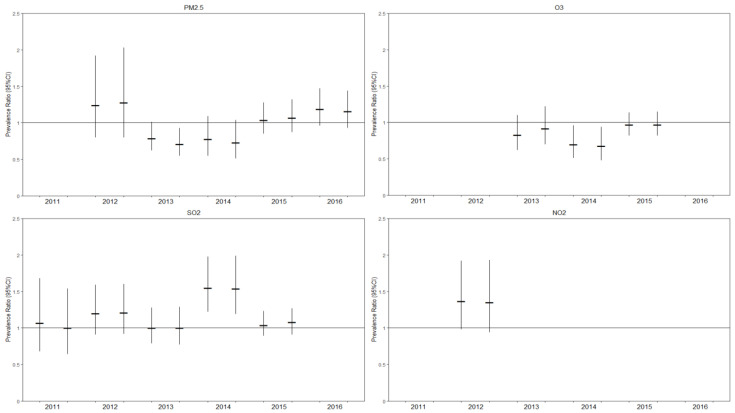
Crude and adjusted estimates of the prevalence ratio between increased outdoor air pollution and depression, with corresponding 95% confidence intervals). The annual crude and adjusted estimates are reported on the left and right, respectively; Potential covariates included: continuous age (centered at 15 years), sex, marital status, total household income, education, employment status, urban versus rural households, cigarette smoking, and chronic illness.

Estimates of the PR (95% CI) and aPR (95% CI) are depicted in Figure 1. Exposure to the upper concentration quartile levels of the four outdoor air pollutants was not shown to be associated with a greater prevalence of MDE or elevated severity of depressive symptoms (Figure 1). No evidence of modification or confounding were found (Data not shown). All final models included adjustment for continuous age (centered at 15 years), sex, marital status, total household income, education, employment status, urban versus rural households, cigarette smoking, and chronic illness (Supplementary Tables S2–S5). Although not all the covariates were included for adjustment each year, due to data availability, a decision made a priori was that a reduction in the precision of the models determined variable exclusion. In instances where the main association between the outdoor air pollutants and depression were shown to be statistically significant (e.g., 2014 O_3_ and SO_2_), these were not interpreted as a meaningful association if they did not occur in more than one year and were considered a Type I error (Supplementary Tables S2–S5). Despite the overall lack of statistical significance, the final adjusted models showed expected associations between the individual-level determinants of depression.

In the models adjusting for neighborhood-level deprivation, social deprivation quintiles 4 and quintile 5 (most deprived), compared to quintile 1 (least deprived), had a statistically significant greater frequency of elevated severity of depressive symptoms (Supplementary Table S6). These results also demonstrate no association between material deprivation quintiles 2, 3, 4, and 5 (most deprived), compared to the least deprived quintile, and depression, as expected.

## 4. Discussion

To our knowledge, this is the first population-based descriptive study using direct measures of depression in nationally representative samples to examine whether living in areas of poor outdoor air quality is associated with major depression. Overall, this study did not find evidence that exposure to increased levels (upper concentration quartile) of PM_2.5_, O_3_, SO_2_, and NO_2_ were associated with a greater prevalence of MDE or elevated depressive symptoms (Figure 1). No evidence of modification or confounding was found among the nine potential explanatory variables. However, the expected associations between the known individual-level determinants, and neighborhood-level social deprivation were observed.

The environmental data provided from CANUE were derived using data synthesis methods and are subject to measurement error. Estimates of the annual average concentrations of outdoor air pollution may have diluted a possible association with depression during short-term events of increased levels of outdoor air pollution (e.g., forest fires). This may be of greater clinical relevance to certain subsets of the population who may have a higher vulnerability to the negative health effects during times of higher outdoor air pollution levels from factors influencing inflammation. Despite the use of two measures of depression, these measures are used in national surveys and are brief compared to the detailed diagnostic interviews typically used to diagnose major depressive disorder in clinical settings [42]. Non-differential misclassification of depression may have biased these estimates towards the null, obscuring associations. It is possible that exposure to increased levels of outdoor air pollution may impact mental well-being and contribute to fatigue, without influencing other symptoms of major depression [62].

A limitation of this study was the range of outdoor air pollution exposure as most of the respondents were not exposed to elevated levels of outdoor air pollution that exceeded concentration levels that are considered safe according to the World Health Organization air quality guidelines (Supplementary Figure S1). This study did not measure indoor air quality, a potential determinant of depression. Despite a lack of evidence of an association at these levels of outdoor air pollution observed in Canada, additional research is still needed, particularly in the absence of a known threshold of outdoor air pollution below which there is no negative impact on health [2]. Additionally, certain characteristics of the built environment may influence exposure to outdoor air pollution. Individuals utilizing active transportation methods such as walking or cycling to school or work, compared to those who commute to work with motorized transportation, may experience higher levels and a longer duration of outdoor air pollution exposure. Beyond a certain threshold, it may be hypothesized that there may be an association with major depression.

The large sample sizes and multiple survey years of the CCHS increased the precision of the PR estimates, and the Statistics Canada population weights enabled inferences of the Canadian household population, highlighting the important strengths of this study. However, these population weights prevented the use of multilevel modeling that would have been able to simultaneously account for both individual-level and neighborhood level determinants. Future research should utilize longitudinal data and multilevel models to examine possible synergistic effects between outdoor air pollution with other built environmental characteristics (e.g., noise pollution, perceptions of neighborhood safety).

## 5. Conclusions

This study did not find evidence that exposure to increased levels of outdoor air pollution is associated with a greater prevalence of MDE or elevated severity of depressive symptoms. Considering the range of outdoor air pollution levels in Canada were below what would typically be classified as unsafe, future research is necessary to monitor these trends overtime and to replicate these findings in other regions where the levels of outdoor air pollution are higher.

## Figures and Tables

**Table 1:** 2016 descriptive characteristics.

Demographic Characteristics	Overall CCHS (*n* ≈ 55,650)	Increased PM_2.5_ (*n* ≈ 8600)	Low PM_2.5_ (*n* ≈ 47,050)	Depression (*n* ≈ 1950)	No Depression (*n* ≈ 25,100)
	% (95% CI)	% (95% CI)	% (95% CI)	% (95% CI)	% (95% CI)
Mean Age ^a^					
	45.4 (45.4, 45.5)	45.1 (45.0, 45.8)	45.4 (45.3, 45.5)	39.9 (38.5, 41.3)	45.0 (44.8, 45.2)
Age Group					
12–24	17.2 (16.8, 17.5)	16.7 (15.6, 17.8)	17.3 (16.8, 17.7)	27.0 (23.3, 30.7)	17.4 (16.7, 18.0)
25+	82.8 (82.5, 83.2)	83.3 (82.2, 84.4)	82.7 (82.3, 83.2)	73.0 (69.3, 76.7)	82.6 (82.0, 83.3)
Sex					
Females	50.7 (50.7, 50.7)	50.4 (49.4, 51.4)	50.8 (50.4, 51.1)	68.0 (64.4, 71.6)	50.0 (49.6, 50.5)
Males	49.3 (49.3, 49.3)	49.6 (48.6, 50.6)	49.2 (48.9, 49.6)	32.0 (28.4, 35.6)	50.0 (49.5, 50.4)
Marital Status					
Married/ Common Law	57.9 (57.3, 58.5)	53.9 (52.3, 55.5)	59.2 (58.5, 59.8)	42.2 (38.2, 46.1)	59.4 (58.5, 60.4)
Widowed/ Separated/ Divorced	12.1 (11.7, 12.5)	13.4 (12.4, 14.4)	11.7 (11.3, 12.1)	13.5 (11.3, 15.7)	10.9 (10.3, 11.5)
Single	30.0 (29.5, 30.5)	32.7 (31.3, 34.1)	29.2 (28.6, 29.7)	44.3 (40.2, 48.5)	29.6 (28.8, 30.5)
Total Household Income (All Sources)					
Quartile 1 (Lowest)	28.7 (28.0, 29.3)	33.9 (32.2, 35.5)	27.0 (26.4, 27.7)	42.1 (38.1, 46.0)	26.0 (25.0, 26.9)
Quartile 2	26.2 (25.6, 26.8)	25.3 (23.8, 26.8)	26.4 (25.8, 27.1)	25.3 (21.9, 28.8)	26.3 (25.4, 27.3)
Quartile 3	25.0 (24.5, 25.6)	22.8 (21.4, 24.2)	25.7 (25.1, 26.4)	23.0 (19.5, 26.4)	25.9 (25.0, 26.8)
Quartile 4 (Highest)	20.1 (19.6, 20.7)	18.0 (16.6, 19.4)	20.8 (20.1, 21.4)	9.63 (7.25, 12.0)	21.8 (20.9, 22.7)
Education ^b^					
Less than High School	18.5 (18.0, 18.9)	16.9 (15.9, 18.0)	18.9 (18.4, 19.4)	24.3 (42.0, 50.2)	16.1 (15.5, 16.7)
High School	23.3 (22.7, 23.9)	21.8 (20.4, 23.2)	23.8 (23.1, 24.4)	29.6 (26.2, 33.0)	23.9 (23.0, 24.8)
Post-Secondary	58.3 (57.6, 58.9)	61.3 (59.6, 63.0)	57.3 (56.6, 58.0)	46.1 (42.0, 50.2)	59.9 (59.0, 60.9)
Employment Status (Past Week) ^c^					
Worked	61.8 (61.1, 62.5)	61.8 (60.0, 63.5)	61.8 (61.1, 62.5)	49.9 (45.7, 54.1)	64.4 (63.3, 65.4)
Absent from job	5.00 (4.70, 5.30)	4.38 (3.60, 5.16)	5.19 (4.86, 5.52)	6.02 (3.93, 8.11)	4.58 (4.12, 5.04)
No job	33.2 (32.6, 33.8)	33.8 (32.2, 35.5)	33.0 (32.3, 33.7)	44.1 (39.9, 48.2)	31.1 (30.1, 32.0)
Geographic Region					
Urban household	82.7 (82.1, 83.3)	97.3 (96.7, 97.9)	78.2 (77.4, 79.0)	85.1 (82.8, 87.3)	81.3 (80.4, 82.2)
Rural	17.3 (16.7, 17.9)	2.73 (2.13, 3.32)	21.8 (21.0, 22.6)	14.9 (12.7, 82.2)	18.7 (17.8, 19.6)
Season of Interview ^d^					
Summer (June–August)	21.0 (20.5, 21.5)	21.1 (19.4, 22.8)	21.0 (20.4, 21.6)	17.0 (14.4, 19.6)	22.5 (21.7, 23.2)
Fall (September–November)	26.7 (26.1, 27.2)	26.6 (24.8, 28.5)	26.7 (26.0, 27.4)	29.7 (25.9, 33.6)	26.9 (26.1, 27.8)
Winter (December–February)	23.9 (23.4, 24.4)	25.1 (23.3, 26.9)	23.6 (22.9, 24.2)	28.0 (24.0, 31.9)	25.0 (24.2, 25.7)
Spring (March–May)	28.4 (28.0, 28.8)	27.1 (25.3, 29.0)	28.8 (28.1, 29.5)	25.3 (21.9, 28.7)	25.6 (25.0, 26.3)
Cigarette Smoking					
Daily/ Occasional	16.8 (16.3, 17.4)	17.1 (15.8, 18.4)	16.8 (16.2, 17.3)	33.7 (29.9, 37.5)	15.6 (14.8, 16.4)
Not at all	83.2 (82.6, 83.7)	82.9 (81.6, 84.2)	83.2 (82.7, 83.8)	66.3 (62.5, 70.1)	84.4 (83.6, 85.2)
Chronic Illness					
At Least One of Ten ^e^	51.4 (50.8, 52.1)	50.5 (48.9, 52.1)	51.7 (51.0, 52.4)	67.1 (63.0, 71.1)	50.0 (49.0, 51.0)
None	48.6 (47.9, 49.2)	49.5 (47.9, 51.1)	48.3 (47.6, 49.0)	32.9 (28.9, 37.0)	50.0 (49.0, 51.0)
Social Deprivation					
Quintile 1 (Least)	26.2 (25.2, 27.2)	20.3 (18.1, 22.5)	28.0 (26.8, 29.1)	22.4 (18.8, 26.0)	31.3 (30.0, 32.7)
Quintile 2	19.4 (18.4, 20.4)	14.1 (12.2, 16.0)	21.1 (20.0, 22.2)	16.2 (12.8, 19.5)	19.4 (18.0, 20.8)
Quintile 3	17.1 (16.1, 18.1)	15.2 (12.9, 17.6)	17.7 (16.6, 18.7)	14.4 (11.9, 17.0)	15.5 (15.2, 17.8)
Quintile 4	21.6 (20.5, 22.6)	26.4 (23.7, 29.1)	20.1 (19.0, 21.2)	22.7 (19.2, 26.2)	19.4 (18.0, 20.8)
Quintile 5 (Most)	15.8 (14.9, 16.6)	23.9 (21.6, 26.2)	13.2 (12.4, 14.0)	24.3 (20.2, 28.3)	13.4 (12.3, 14.4)
Material Deprivation					
Quintile 1 (Least)	26.3 (25.3, 27.3)	27.1 (24.5, 29.8)	26.0 (24.9, 27.1)	22.2 (17.9, 26.6)	24.3 (22.9, 25.7)
Quintile 2	21.2 (20.1, 22.2)	18.5 (16.0, 21.0)	22.0 (20.8, 23.2)	16.8 (13.7, 20.0)	21.9 (20.4, 23.4)
Quintile 3	21.4 (20.3, 22.5)	16.9 (14.5, 19.2)	22.8 (21.6, 24.1)	22.1 (18.4, 25.7)	21.2 (19.7, 22.7)
Quintile 4	14.0 (13.1, 15.0)	15.5 (13.1, 17.9)	13.6 (12.6, 14.6)	15.4 (12.6, 18.1)	14.1 (12.8, 15.4)
Quintile 5 (Most)	17.1 (16.2, 18.0)	22.0 (19.6, 24.3)	15.6 (14.6, 16.6)	23.5 (19.8, 27.2)	18.5 (17.1, 19.9)

Sample sizes rounded to base 50 as per Statistics Canada guidelines and may not add up; ^a^ In accordance with Statistics Canada guidelines, and the 95% CI corresponding to the weighted estimate for mean age is reported instead of the standard deviation; ^b^ Only asked to respondents 15 years old and older; ^c^ Only asked to respondents aged 15–75; ^d^ Season of interview was not included in the subsequent models due to nearly identical frequencies in exposure groups for all four outdoor air pollutants; ^e^ Includes: arthritis, asthma, back problems, chronic bronchitis, COPD, diabetes, cardiovascular disease, high blood pressure, migraine, and stroke.

## Data Availability

The use of the annual Canadian Community Health Survey microdata files required all data analyses for this study to be conducted in the Prairie Regional Research Data Centre (PRRDC) located in the Taylor Family Digital Library at the University of Calgary. Access, sharing, and use of CANUE metadata files follows the agreement of intended use.

## Supplementary Materials

The following are available online at https://www.mdpi.com/1660-4601/18/5/2450/s1, Table S1: Annual sample characteristics from 2011 to 2016; Figure S1: Annual average concentration of PM_2.5_, O_3_, SO_2_, and NO_2_; Table S2: Adjusted models of increased PM_2.5_ and depression; Table S3: Adjusted models of increased O_3_ and depression; Table S4: Adjusted models of increased SO_2_ and depression; Table S5: Adjusted models of increased NO_2_ and depression; Table S6: Adjusted models of social, and material deprivation and depression in 2016.
