# Supplementary material for: Outdoor Air Pollution and Depression in Canada: A Population-Based Cross-Sectional Study from 2011 to 2016
Source: Int J Environ Res Public Health. 2021 Mar 2;18(5):2450. doi: 10.3390/ijerph18052450 (PMC7967582; doi:10.3390/ijerph18052450)
Supplement: Supplementary file 1 [file ijerph-18-02450-s001.zip › ijerph 1109557_Supplementary/ijerph 1109557_Supplementary Fig_2021_Mar 1.pptx]

## Slide 1
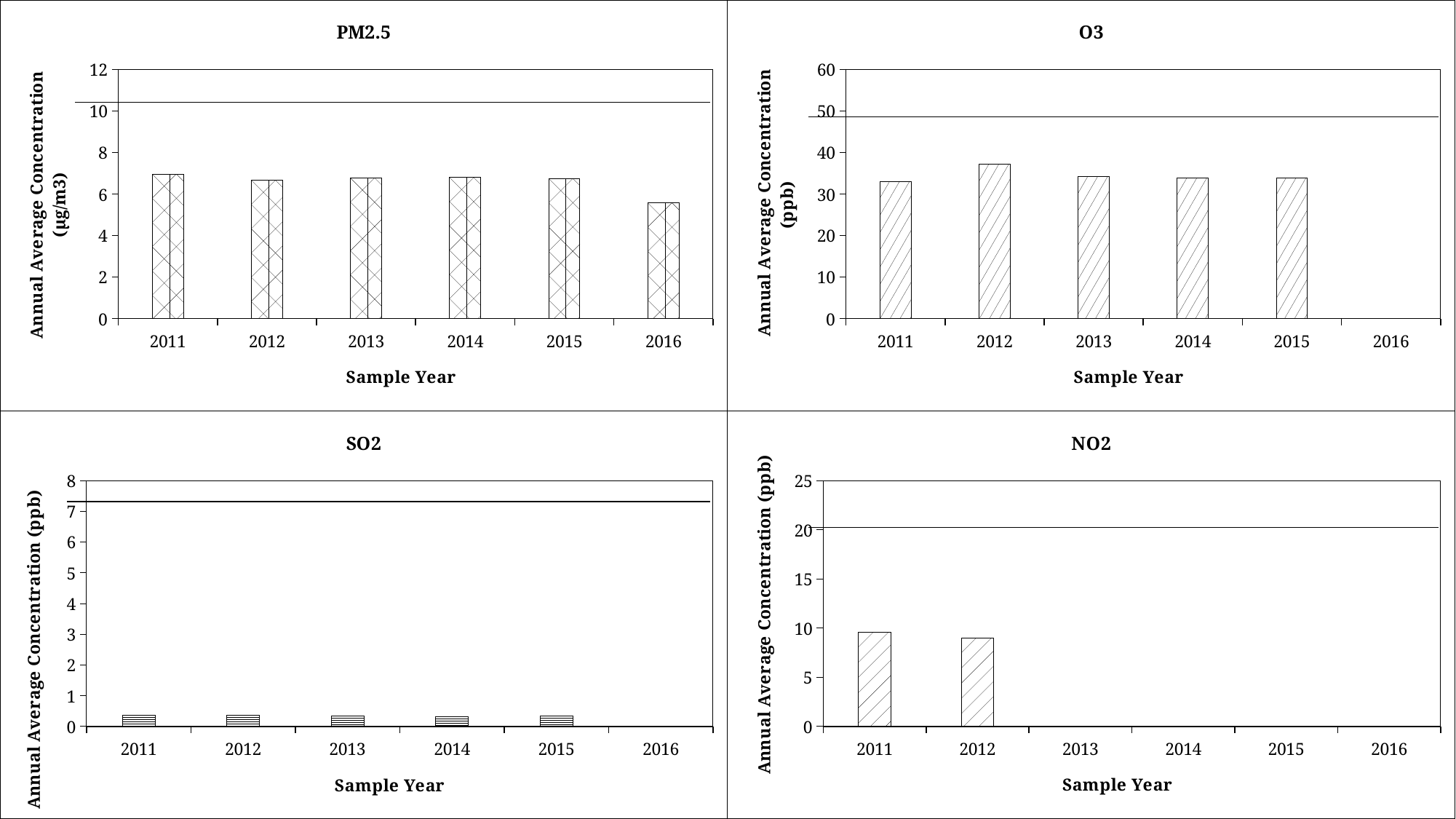

### Chart: PM2.5
| Category | Overall PM2.5 |
|---|---|
| 2011 | 6.958961 |
| 2012 | 6.659712 |
| 2013 | 6.770024 |
| 2014 | 6.794602 |
| 2015 | 6.742607 |
| 2016 | 5.570019 |
### Chart: O3
| Category | Overall O3 |
|---|---|
| 2011 | 33.084095 |
| 2012 | 37.173296 |
| 2013 | 34.303644 |
| 2014 | 33.938657 |
| 2015 | 33.924766 |
| 2016 | None |
### Chart: SO2
| Category | Overall SO2 |
|---|---|
| 2011 | 0.359631 |
| 2012 | 0.358821 |
| 2013 | 0.331231 |
| 2014 | 0.314052 |
| 2015 | 0.34177 |
| 2016 | None |
### Chart: NO2
| Category | Overall NO2 |
|---|---|
| 2011 | 9.592967 |
| 2012 | 9.013734 |
| 2013 | None |
| 2014 | None |
| 2015 | None |
| 2016 | None |
