# Supplementary material for: Outdoor Air Pollution and Depression in Canada: A Population-Based Cross-Sectional Study from 2011 to 2016
Source: Int J Environ Res Public Health. 2021 Mar 2;18(5):2450. doi: 10.3390/ijerph18052450 (PMC7967582; doi:10.3390/ijerph18052450)
Supplement: Supplementary file 1 [file ijerph-18-02450-s001.zip › ijerph 1109557_Supplementary/Supplementary Tables_ijerph 1109557_2021_Mar 1.docx]

**Table S1.** Annual sample characteristics from 2011 to 2016.

| **Survey Year** | **Total CCHS** | **Response Rate (%)** | **Overall Sample Year ^a^** | **Missing Depression ^b^** | **Missing Exposure (%)** | **Study Population ^c^** | **Provinces** |
| --- | --- | --- | --- | --- | --- | --- | --- |
| 2011 | *N* ≈ 63,550 | 69.8% | *n* ≈ 63,400 | *n* ≈ 52,550 | SO_2_ (16%) | SO_2_ (*n* ≈ 10,200) | NL, PE, NS, NB, SK, NU |
| 2012 | *N* ≈ 62,100 | 67.0% | *n* ≈ 62,050 | *n* ≈ 46,050 | PM_2.5_ (<1%)  SO_2_ (12%)  NO_2_ (<1%) | PM_2.5_ (*n* ≈ 15,900)  SO_2_ (*n* ≈ 14,200)  NO_2_ (*n* ≈ 15,950) | NL, PE, NS, NB, SK, AB, NU |
| 2013 | *N* ≈ 64,350 | 66.8% | *n* ≈ 63,300 | *n* ≈ 43,350 | PM_2.5_ (<1%)  O_3_ (<1%)  SO_2_ (8%) | PM_2.5_ (*n* ≈ 20,750)  O_3_ (*n* ≈ 20,950)  SO_2_ (*n* ≈ 20,650) | NL, PE, NS, QB, MB, NT, NU |
| 2014 | *N* ≈ 63,950 | 65.6% | *n* ≈ 63,850 | *n* ≈ 42,950 | PM_2.5_ (<1%)  O_3_ (<1%)  SO_2_ (10%) | PM_2.5_ (*n* ≈ 20,700)  O_3_ (*n* ≈ 20,900)  SO_2_ (*n* ≈ 20,150) | NL, PE, NS, QB, MB, NT, NU |
| 2015 | *N* ≈ 51,550 | 57.5% | *n* ≈ 51,550 | *n* ≈ 20,250 | PM_2.5_ (<1%)  O_3_ (<1%)  SO_2_ (7%) | PM_2.5_ (*n* ≈ 31,250)  O_3_ (*n* ≈ 31,250)  SO_2_ (*n* ≈ 28,400) | NL, PE, NS, NB, ON, MB, SK, BC |
| 2016 | *N* ≈ 55,700 | 61.3% | *n* ≈ 55,650 | *n* ≈ 28,600 | PM_2.5_ (<1%)  SD (<1%)  MD (<1%) | PM_2.5_ (*n* ≈ 27,050)  SD (*n* ≈ 26,000)  MD (*n* ≈ 26,000) | NL, PE, NS, NB, ON, MB, SK |

Sample sizes rounded to base 50 as per Statistics Canada guidelines and may not add up; **^a^** Excluded respondents with missing 6-digit postal code match in the CANUE datasets; **^b^** Excluded respondents who did not complete the depression module, proxy interviews, and those who had incomplete responses to the depression questions; ^c^ Bivariate sample sizes between each outdoor air pollutant, social, and material deprivation with depression; Provinces: NL = Newfoundland; PE = Prince Edward Island; NS = Nova Scotia; NB = New Brunswick; ON = Ontario; QB = Quebec; MB = Manitoba; SK = Saskatchewan; AB = Alberta; BC = British Columbia; NT = Northwest Territories; NU = Nunavut Territories; YT = Yukon Territories.

**Table S2.** Adjusted models of increased PM_2.5_ and depression.

|  | **2012** | **2013** | **2014** | **2015** | **2016** |
| --- | --- | --- | --- | --- | --- |
|  | aPR (95% CI) | aPR (95% CI) | aPR (95% CI) | aPR (95% CI) | aPR (95% CI) |
| PM_2.5_ | 1.28 (0.80, 2.03)  *p* = 0.305 | 0.71 (0.55, 0.93)  *p* = 0.013 | 0.73 (0.51, 1.04)  *p* = 0.082 | 1.07 (0.87, 1.32)  *p* = 0.533 | 1.16 (0.93, 1.44)  *p* = 0.193 |
| Age ^a^ | 0.98 (0.97, 0.98)  *p* <0.001 | 0.98 (0.97, 0.98)  *p* <0.001 | 0.99 (0.98, 0.99)  *p* <0.001 | 0.98 (0.97, 0.98)  *p* <0.001 | 0.98 (0.97, 0.98)  *p* <0.001 |
| Female | 1.84 (1.43, 2.35)  *p* <0.001 | 1.91 (1.50, 2.41)  *p* <0.001 | 1.80 (1.44, 2.24)  *p* <0.001 | 1.55 (1.30, 1.83)  *p* <0.001 | 2.22 (1.85, 2.67)  *p* <0.001 |
| Marital Status |  |  |  |  |  |
| Married/ Common  Law | * | *Ref* | *Ref* | *Ref* | *Ref* |
| Widowed/  Separated/  Divorced |  | 2.12 (1.50, 3.00)  *p* <0.001 | 1.58 (1.15, 2.16)  *p* = 0.050 | 1.70 (1.36, 2.14)  *p* <0.001 | 1.31 (1.05, 1.65)  *p* = 0.019 |
| Single |  | 1.52 (1.15, 2.01)  *p* = 0.040 | 1.76 (1.33, 2.32)  *p* <0.001 | 1.41 (1.14, 1.74)  *p* = 0.001 | 1.38 (1.07, 1.78)  *p* = 0.014 |
| Total Household Income |  |  |  |  |  |
| Quartile 1 (Lowest) | 1.52 (1.20, 1.93)  *p* <0.001 | 1.53 (1.21, 1.94)  *p* <0.001 | *Ref* | *Ref* | *Ref* |
| Quartile 2 | * | * | 0.93 (0.68, 1.27)  *p* = 0.656 | 0.66 (0.54, 0.80)  *p* <0.001 | 0.73 (0.60, 0.89)  *p* = 0.002 |
| Quartile 3 |  |  | 0.74 (0.57, 0.97)  *p* = 0.030 | 0.56 (0.45, 0.69)  *p* <0.001 | 0.71 (0.57, 0.88)  *p* = 0.002 |
| Quartile 4 (Highest) |  |  | 0.61 (0.36, 1.02)  *p* = 0.058 | 0.31 (0.23, 0.43)  *p* <0.001 | 0.38 (0.28, 0.52)  *p* <0.001 |
| Education ^b^ |  |  |  |  |  |
| Post-Secondary | * | *Ref* | *Ref* | *Ref* | *Ref* |
| High School |  | 0.93 (0.70, 1.24)  *p* = 0.611 | 0.96 (0.75, 1.21)  *p* = 0.714 | 1.13 (0.93, 1.37)  *p* = 0.213 | 1.27 (1.05, 1.54)  *p* = 0.015 |
| Less than High  School |  | 0.93 (0.72, 1.21)  *p* = 0.595 | 0.71 (0.53, 0.95)  *p* = 0.020 | 1.06 (0.85, 1.32)  *p* = 0.613 | 1.41 (1.11, 1.79)  *p* = 0.005 |
| Employment Status  (Past Week) ^c^ |  |  |  |  |  |
| Worked | * | *Ref* | * | * | * |
| Absent from job |  | 1.51 (0.94, 2.44)  *p* = 0.089 |  |  |  |
| No job |  | 1.23 (0.99, 1.54)  *p* = 0.065 |  |  |  |
| Urban | * | 1.11 (0.90, 1.37)  *p* = 0.324 | * | 1.03 (0.88, 1.20)  *p* = 0.727 | 1.31 (1.09, 1.58)  *p* = 0.040 |
| Cigarette Smoking ^d^ | 2.00 (1.61, 2.49)  *p* <0.001 | 1.83 (1.45, 2.32)  *p* <0.001 | 2.57 (2.02, 3.28)  *p* <0.001 | 1.90 (1.60, 2.25)  *p* <0.001 | 2.51 (2.08, 3.02)  *p* <0.001 |
| Chronic Illness ^e^ | 2.26 (1.77, 2.90)  *p* <0.001 | 2.18 (1.73, 2.74)  *p* <0.001 | 2.09 (1.65, 2.63)  *p* <0.001 | 3.80 (3.15, 4.59)  *p* <0.001 | 2.71 (2.20, 3.33)  *p* <0.001 |

**^a^** Continuous age centered at 15 years; ^b^ Only asked to respondents 15 years old and older; **^c^** Only asked to respondents aged 15-75; **^d^** Daily/ occasional versus never; ^e^ At least 1 of 10 self-reported professional diagnosis of chronic illnesses that include: arthritis, asthma, back problems, chronic bronchitis, COPD, diabetes, cardiovascular disease, high blood pressure, migraine, and stroke.

**Table S3.** Adjusted models of increased O_3_ and depression.

|  | **2013** | **2014** | **2015** |
| --- | --- | --- | --- |
|  | aPR (95% CI) | aPR (95% CI) | aPR (95% CI) |
| O_3_ | 0.92 (0.70, 1.22)  *p* = 0.565 | 0.68 (0.48, 0.94)  *p* = 0.021 | 0.97 (0.82, 1.15)  *p* = 0.703 |
| Age ^a^ | 0.98 (0.97, 0.98)  *p* <0.001 | 0.98 (0.98, 0.99)  *p* <0.001 | 0.98 (0.97, 0.98)  *p* <0.001 |
| Female | 1.97 (1.57, 2.48)  *p* <0.001 | 1.76 (1.40, 2.20)  *p* <0.001 | 1.55 (1.30, 1.84)  *p* <0.001 |
| Marital Status |  |  |  |
| Married/ Common  Law | *Ref* | *Ref* | *Ref* |
| Widowed/  Separated/  Divorced | 1.98 (1.41, 2.77)  *p* <0.001 | 1.67 (1.21, 2.32)  *p* = 0.002 | 1.71 (1.36, 2.15)  *p* <0.001 |
| Single | 1.38 (1.05, 1.83)  *p* = 0.022 | 1.86 (1.39, 2.49)  *p* <0.001 | 1.41 (1.14, 1.75)  *p* = 0.010 |
| Total Household Income |  |  |  |
| Quartile 1 (Lowest) | 1.63 (1.30, 2.04)  *p* <0.001 | 1.14 (0.89, 1.45)  *p* = 0.293 | *Ref* |
| Quartile 2 | * | * | 0.66 (0.55, 0.81)  *p* <0.001 |
| Quartile 3 |  |  | 0.56 (0.45, 0.70)  *p* <0.001 |
| Quartile 4 (Highest) |  |  | 0.31 (0.23, 0.43)  *p* <0.001 |
| Education ^b^ |  |  |  |
| Post-Secondary | *Ref* | *Ref* | *Ref* |
| High School | 0.94 (0.71, 1.24)  *p* = 0.657 | 0.93 (0.72, 1.20)  *p* = 0.575 | 1.13 (0.93, 1.37)  *p* = 0.212 |
| Less than High  School | 0.81 (0.63, 1.03)  *p* = 0.091 | 0.76 (0.55, 1.06)  *p* = 0.109 | 1.06 (0.85, 1.32)  *p* = 0.605 |
| Employment Status  (Past Week) ^c^ |  |  |  |
| Worked | * | *Ref* | * |
| Absent from job |  | 1.82 (1.28, 2.59)  *p* = 0.001 |  |
| No job |  | 1.75 (1.36, 2.25)  *p* <0.001 |  |
| Urban | 0.94 (0.77, 1.14)  *p* = 0.513 | 0.93 (0.75, 1.16)  *p* = 0.528 | 1.04 (0.89, 1.21)  *p* = 0.635 |
| Cigarette Smoking ^d^ | 2.01 (1.60, 2.54)  *p* <0.001 | 2.52 (1.96, 3.24)  *p* <0.001 | 1.89 (1.60, 2.24)  *p* <0.001 |
| Chronic Illness ^e^ | 2.22 (1.77, 2.78)  *p* <0.001 | 2.03 (1.61, 2.56)  *p* <0.001 | 3.80 (3.14, 4.58)  *p* <0.001 |

**^a^** Continuous age centered at 15 years; ^b^ Only asked to respondents 15 years old and older; **^c^** Only asked to respondents aged 15-75; **^d^** Daily/ occasional, and never; ^e^ At least 1 of 10 self-reported professional diagnosis of chronic illnesses that include: arthritis, asthma, back problems, chronic bronchitis, COPD, diabetes, cardiovascular disease, high blood pressure, migraine, and stroke.

**Table S4.** Adjusted models of increased SO_2_ and depression.

|  | **2011** | **2012** | **2013** | **2014** | **2015** |
| --- | --- | --- | --- | --- | --- |
|  | aPR (95% CI) | aPR (95% CI) | aPR (95% CI) | aPR (95% CI) | aPR (95% CI) |
| SO_2_ | 1.00 (0.64, 1.54)  *p* = 0.985 | 1.21 (0.92, 1.60)  *p* = 0.173 | 1.00 (0.77, 1.29)  *p* = 0.979 | 1.54 (1.19, 1.99)  *p* = 0.001 | 1.08 (0.91, 1.27)  *p* = 0.404 |
| Age ^a^ | 0.97 (0.96, 0.98)  *p* <0.001 | 0.98 (0.97, 0.98)  *p* <0.001 | 0.98 (0.97, 0.98)  *p* <0.001 | 0.98 (0.98, 0.99)  *p* <0.001 | 0.98 (0.97, 0.98)  *p* <0.001 |
| Female | 2.08 (1.59, 2.72)  *p* <0.001 | 1.75 (1.35, 2.27)  *p* <0.001 | 1.89 (1.50, 2.40)  *p* <0.001 | 1.72 (1.36, 2.17)  *p* <0.001 | 1.54 (1.30, 1.83)  *p* <0.001 |
| Marital Status |  |  |  |  |  |
| Married/ Common  Law | *Ref* |  | *Ref* | *Ref* | *Ref* |
| Widowed/  Separated/  Divorced | 1.65 (1.13, 2.40)  *p* = 0.009 | * | 2.04 (1.45, 2.87)  *p* <0.001 | 1.61 (1.16, 2.23)  *p* = 0.005 | 1.59 (1.30, 1.95)  *p* <0.001 |
| Single | 0.88 (0.61, 1.25)  *p* = 0.470 |  | 1.49 (1.13, 1.97)  *p* = 0.004 | 1.82 (1.35, 2.44)  *p* <0.001 | 1.40 (1.14, 1.74)  *p* = 0.002 |
| Total Household Income |  |  |  |  |  |
| Quartile 1 (Lowest) | 1.39 (1.05, 1.83)  *p* = 0.009 | *Ref* | *Ref* | *Ref* | *Ref* |
| Quartile 2 | * | 0.76 (0.58, 1.00)  *p* = 0.051 | 0.70 (0.53, 0.93)  *p* = 0.013 | 0.94 (0.68, 1.31)  *p* = 0.731 | 0.67 (0.55, 0.81)  *p* <0.001 |
| Quartile 3 |  | 0.61 (0.46, 0.82)  *p* = 0.001 | 0.68 (0.51, 0.90)  *p* = 0.006 | 0.85 (0.65, 1.12)  *p* = 0.253 | 0.50 (0.41, 0.60)  *p* <0.001 |
| Quartile 4 (Highest) |  | 0.50 (0.32, 0.77)  *p* = 0.002 | 0.38 (0.23, 0.61)  *p* <0.001 | 0.71 (0.41, 1.24)  *p* = 0.231 | 0.30 (0.22, 0.42)  *p* <0.001 |
| Education ^b^ |  |  |  |  |  |
| Post-Secondary | * | * | *Ref* | *Ref* | *Ref* |
| High School |  |  | 0.92 (0.69, 1.23)  *p* = 0.583 | 0.91 (0.70, 1.18)  *p* = 0.487 | 1.15 (0.94, 1.39)  *p* = 0.178 |
| Less than High  School |  |  | 0.96 (0.74, 1.24)  *p* = 0.744 | 0.76 (0.54, 1.07)  *p* = 0.113 | 1.03 (0.84, 1.26)  *p* = 0.775 |
| Employment Status  (Past Week) ^c^ |  |  |  |  |  |
| Worked | * | * | *Ref* | *Ref* | * |
| Absent from job |  |  | 1.48 (0.91, 2.42)  *p* = 0.114 | 1.93 (1.35, 2.74)  *p* <0.001 |  |
| No job |  |  | 1.19 (0.95, 1.49)  *p* = 0.121 | 1.79 (1.39, 2.30)  *p* <0.001 |  |
| Urban | 1.19 (0.93, 1.51)  *p* = 0.165 | 1.49 (1.18, 1.88)  *p* = 0.001 | 0.98 (0.80, 1.20)  *p* = 0.836 | 1.06 (0.85, 1.31)  *p* = 0.609 | 0.99 (0.84, 1.17)  *p* = 0.937 |
| Cigarette Smoking ^d^ | 2.45 (1.86, 3.22)  *p* <0.001 | 2.01 (1.59, 2.53)  *p* <0.001 | 1.80 (1.42, 2.28)  *p* <0.001 | 2.53 (1.96, 3.28)  *p* <0.001 | 1.94 (1.62, 2.31)  *p* <0.001 |
| Chronic Illness ^e^ | 2.10 (1.58, 2.79)  *p* <0.001 | 2.20 (1.70, 2.84)  *p* <0.001 | 2.19 (1.74, 2.75)  *p* <0.001 | 1.99 (1.56, 2.53)  *p* <0.001 | 3.62 (2.98, 4.39)  *p* <0.001 |

**^a^** Continuous age centered at 15 years; ^b^ Only asked to respondents 15 years old and older; **^c^** Only asked to respondents aged 15-75; **^d^** Daily/ occasional, and never; ^e^ At least 1 of 10 self-reported professional diagnosis of chronic illnesses that include: arthritis, asthma, back problems, chronic bronchitis, COPD, diabetes, cardiovascular disease, high blood pressure, migraine, and stroke.

**Table S5.** Adjusted models of increased NO_2_ and depression.

|  | **2012** |
| --- | --- |
|  | aPR (95% CI) |
| NO_2_ | 1.35 (0.94, 1.93)  *p* = 0.103 |
| Age ^a^ | 0.97 (0.97, 0.98)  *p* <0.001 |
| Female | 1.79 (1.40, 2.28)  *p* <0.001 |
| Marital Status |  |
| Married/ Common  Law | *Ref* |
| Widowed/  Separated/  Divorced | 1.95 (1.36, 2.79)  *p* <0.001 |
| Single | 1.27 (0.92, 1.74)  *p* = 0.141 |
| Total Household Income |  |
| Quartile 1 (Lowest) | 1.38 (1.07, 1.77)  *p* = 0.013 |
| Quartile 2 | * |
| Quartile 3 |  |
| Quartile 4 (Highest) |  |
| Education ^b^ |  |
| Post-Secondary | *Ref* |
| High School | 0.89 (0.69, 1.15)  *p* = 0.371 |
| Less than High  School | 0.92 (0.70, 1.22)  *p* = 0.574 |
| Employment Status  (Past Week) ^c^ |  |
| Worked | * |
| Absent from job |  |
| No job |  |
| Urban | * |
| Cigarette Smoking ^d^ | 1.94 (1.56, 2.42)  *p* <0.001 |
| Chronic Illness ^e^ | 2.30 (1.79, 2.95)  *p* <0.001 |

**^a^** Continuous age centered at 15 years; ^b^ Only asked to respondents 15 years old and older; **^c^** Only asked to respondents aged 15-75; **^d^** Daily/ occasional, and never; ^e^ At least 1 of 10 self-reported professional diagnosis of chronic illnesses that include: arthritis, asthma, back problems, chronic bronchitis, COPD, diabetes, cardiovascular disease, high blood pressure, migraine, and stroke.

**Table S6.** Adjusted models of social, and material deprivation and depression in 2016.

| **2016** | **Social Deprivation** | **Material Deprivation** |
| --- | --- | --- |
|  | aPR (95% CI) | aPR (95% CI) |
| **PM_2.5_** | 1.07 (0.85, 1.34)  *p* = 0.580 | 1.10 (0.88, 1.39)  *p* = 0.406 |
| **Age ^a^** | 0.99 (0.98, 0.99)  *p* <0.001 | 0.99 (0.98, 0.99)  *p* <0.001 |
| **Female** | 2.17 (1.81, 2.61)  *p* <0.001 | 2.18 (1.82, 2.61)  *p* <0.001 |
| **Marital Status** |  |  |
| Married/ Common  Law | *Ref* | *Ref* |
| Widowed/  Separated/  Divorced | 1.30 (1.02, 1.65)  *p* = 0.031 | 1.33 (1.05, 1.68)  *p* = 0.016 |
| Single | 1.42 (1.09, 1.85)  *p* = 0.009 | 1.48 (1.13, 1.94)  *p* = 0.005 |
| **Total Household Income** |  |  |
| Quartile 1 (Lowest) | *Ref* | *Ref* |
| Quartile 2 | 0.69 (0.55, 0.85)  *p* = 0.001 | 0.66 (0.53, 0.82)  *p* <0.001 |
| Quartile 3 | 0.65 (0.51, 0.81)  *p* <0.001 | 0.60 (0.47, 0.75)  *p* <0.001 |
| Quartile 4 (Highest) | 0.33 (0.24, 0.45)  *p* <0.001 | 0.29 (0.21, 0.41)  *p* <0.001 |
| **Urban** | 1.02 (0.84, 1.25)  *p* = 0.830 | 1.16 (0.97, 1.40)  *p* = 0.112 |
| **Deprivation Quintile** |  |  |
| Quintile 1 (Least) | *Ref* | *Ref* |
| Quintile 2 | 1.12 (0.82, 1.51)  *p* = 0.475 | 0.79 (0.59, 1.06)  *p* = 0.121 |
| Quintile 3 | 1.07 (0.83, 1.38)  *p* = 0.619 | 1.03 (0.76, 1.39)  *p* = 0.837 |
| Quintile 4 | 1.34 (1.03, 1.76)  *p* = 0.032 | 1.02 (0.76, 1.38)  *p* = 0.876 |
| Quintile 5 (Most) | 1.88 (1.40, 2.51)  *p* <0.001 | 1.05 (0.77, 1.42)  *p* = 0.762 |

**^a^** Continuous age centered at 15 years.
